# Supplementary material for: Fire weather effects on flammability of indigenous and invasive alien plants in coastal fynbos and thicket shrublands (Cape Floristic Region)
Source: PeerJ. 2020 Nov 11;8:e10161. doi: 10.7717/peerj.10161 (PMC7666561; doi:10.7717/peerj.10161)
Supplement: Supplemental Information 1 — Photos by Tineke Kraaij unless otherwise indicated. [file peerj-08-10161-s001.pdf]

Supplemental Figure S1

Collage of photos showing the different vegetation types, i.e. Fynbos, Thicket, and Invasive Alien Plants (IAP), along the southern Cape coast of South Africa. Photo credits: Tineke Kraaij, except fourth image from the top credited to Tiaan Strydom.

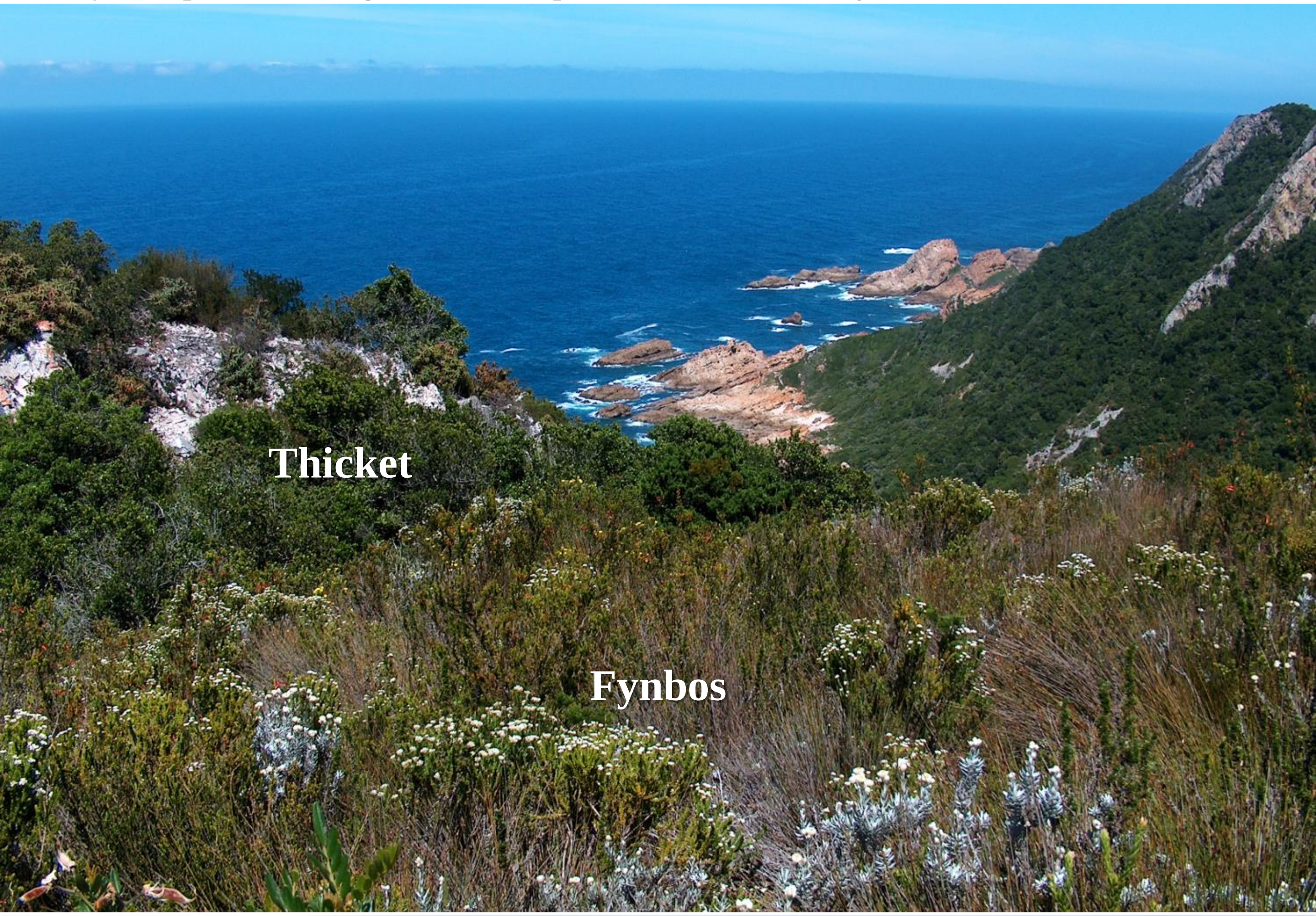

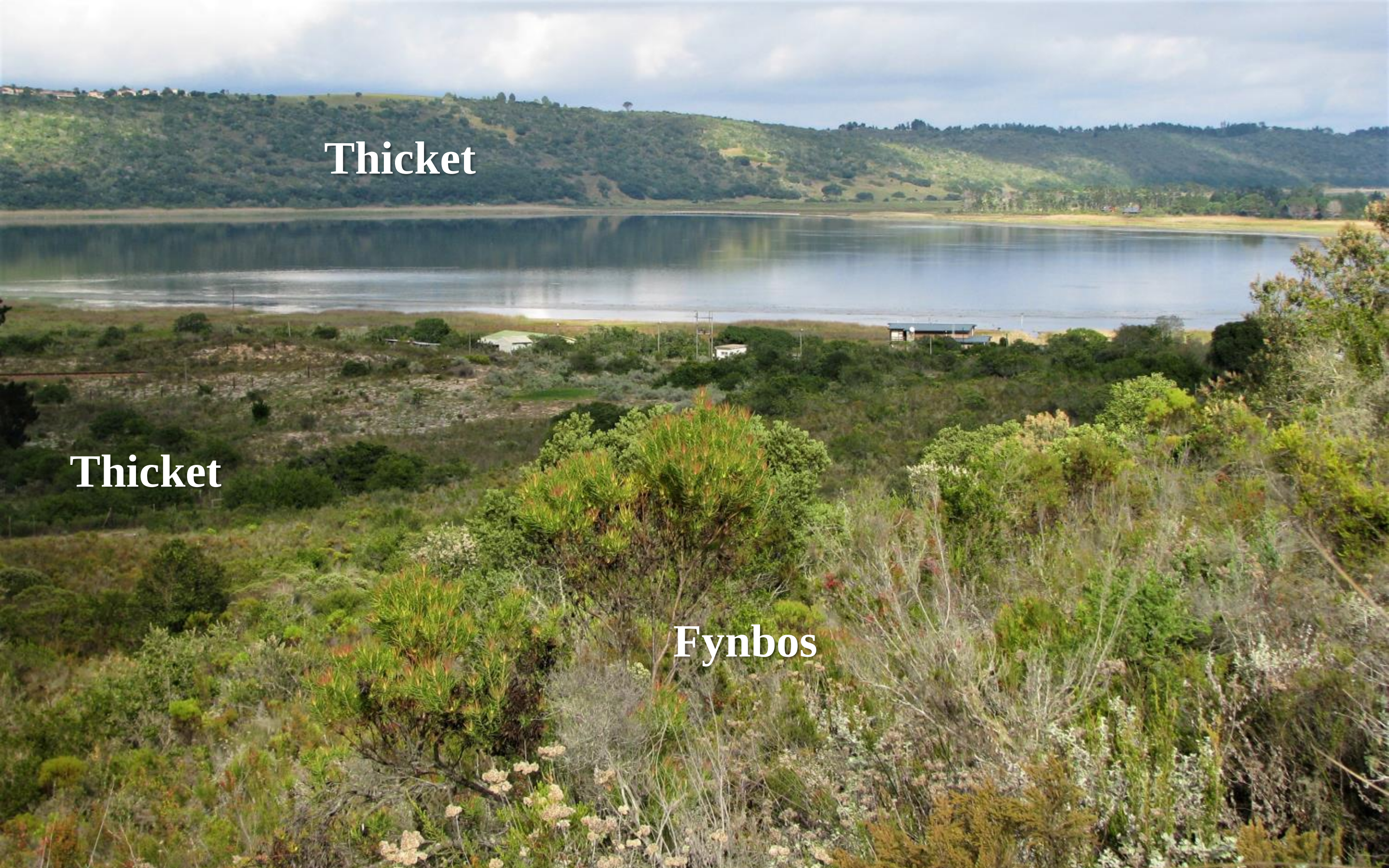

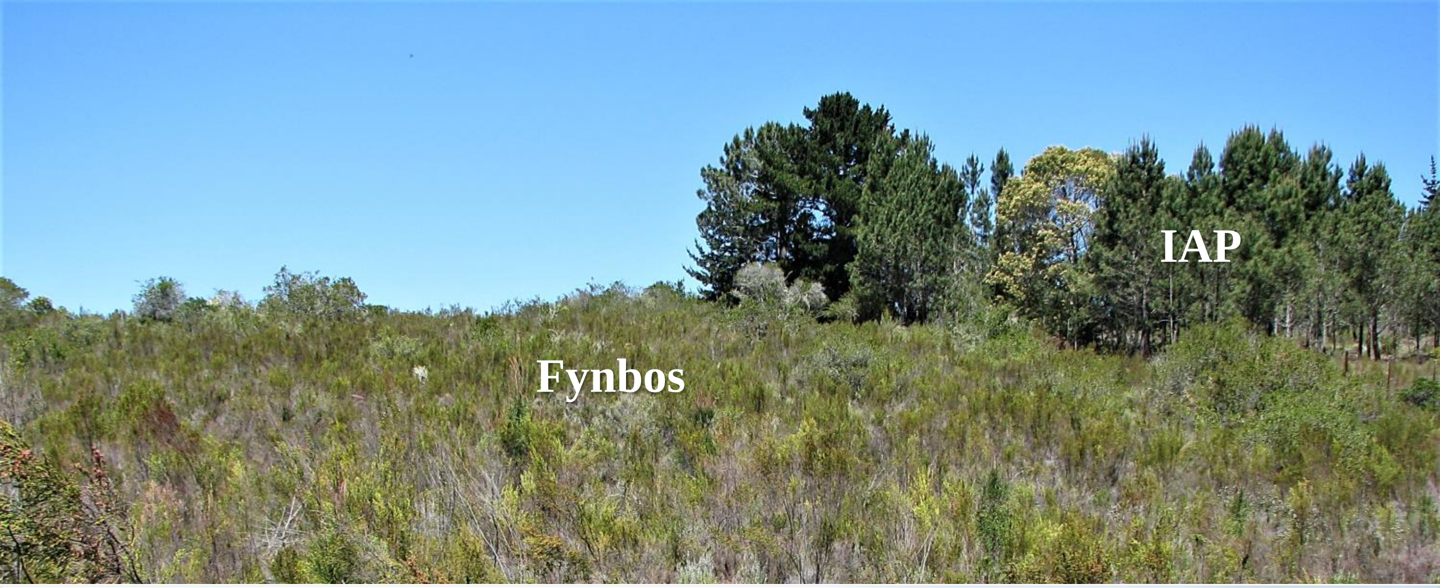
IAP
Fynbos

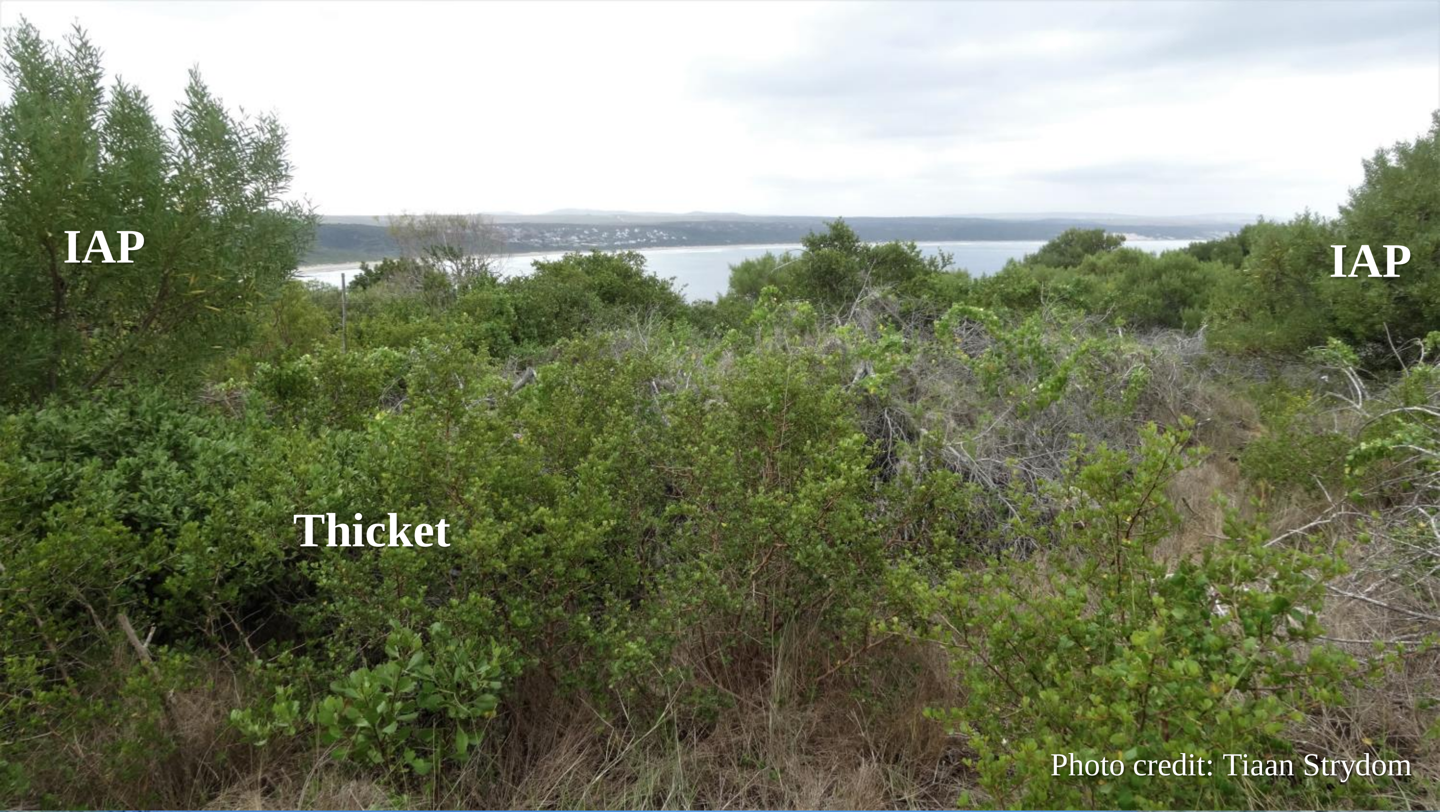
IAP
IAP
Thicket
Photo credit: Tiaan Strydom

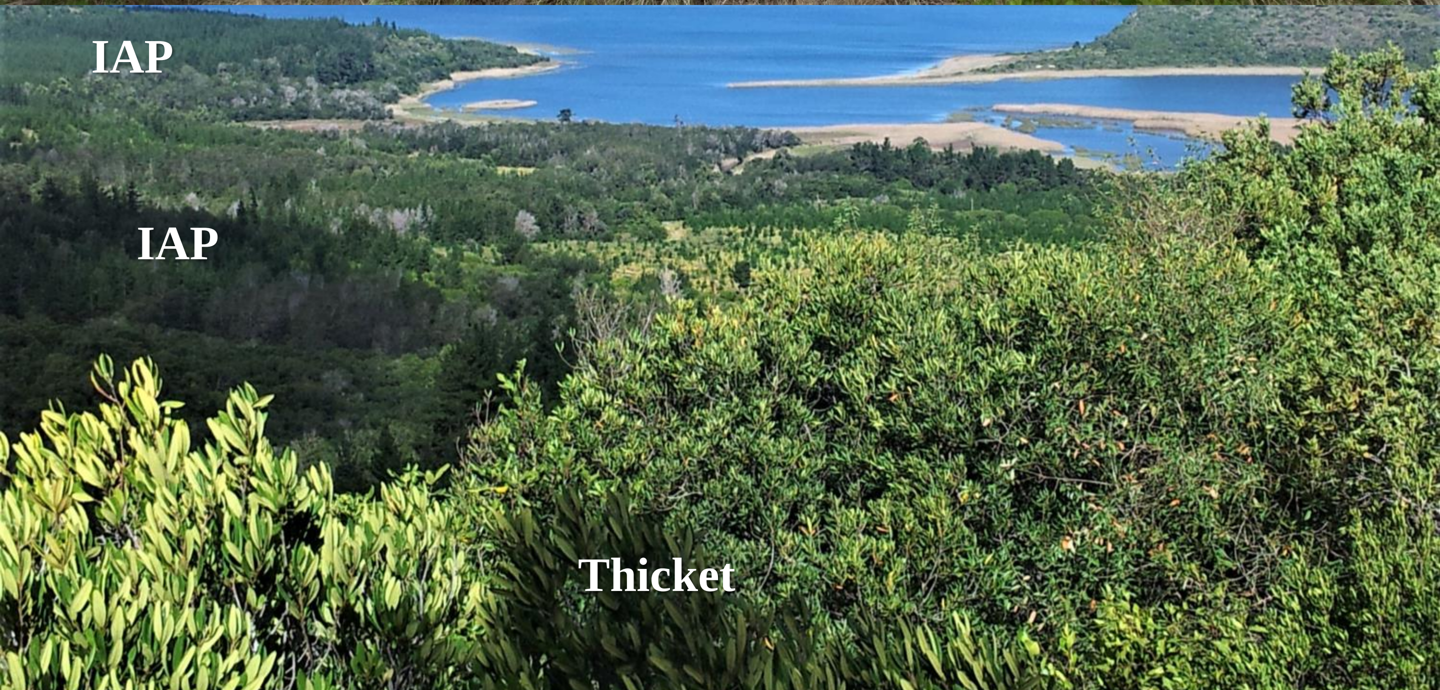
IAP
IAP
Thicket
