# Supplementary material for: Fire weather effects on flammability of indigenous and invasive alien plants in coastal fynbos and thicket shrublands (Cape Floristic Region)
Source: PeerJ. 2020 Nov 11;8:e10161. doi: 10.7717/peerj.10161 (PMC7666561; doi:10.7717/peerj.10161)
Supplement: Supplemental Information 2 — Nomenclature follows The Plant List (2013). [file peerj-08-10161-s002.docx]

Supplemental Table S1

Spearman's rank correlation (rho) results between predictor variables, fire weather and fuel moisture, for each respective species. Nomenclature follows The Plant List (2013).

| No. | Species names | Slope | rho | p |
| --- | --- | --- | --- | --- |
| Fynbos | | | | |
| 1 | *Passerina rigida* Wikstr. | negative | 0.003 | 0.993 |
| 2 | *Erica discolor* Andrews | negative | 0.174 | 0.460 |
| 3 | *Erica canaliculata* Andrews | positive | 0.181 | 0.432 |
| 4 | *Cliffortia ericifolia* E.Mey. ex Harv | positive | 0.114 | 0.621 |
| 5 | *Cliffortia ilicifolia* L. | negative | 0.051 | 0.828 |
| 6 | *Agathosma ovata* (Thunb.) Pillans | negative | 0.210 | 0.358 |
| 7 | *Metalasia muricata* (L.) D.Don | positive | 0.419 | 0.060 |
| 8 | *Phylica axillaris* Lam. | positive | 0.353 | 0.117 |
| 9 | *Aspalathus spinosa* L. | negative | 0.370 | 0.099 |
| 10 | *Leucadendron eucalyptifolium* H. Buek ex Meisn. | positive | 0.070 | 0.763 |
| Thicket | | | | |
| 11 | *Pterocelastrus tricuspidatus* Walp. | negative | 0.097 | 0.674 |
| 12 | *Searsia lucida* (L.) F.A.Barkley | negative | 0.134 | 0.562 |
| 13 | *Tarchonanthus littoralis* P.P.J.Herman | negative | 0.078 | 0.737 |
| 14 | *Diospyros dichrophylla (*Gand.) De Winter | negative | 0.084 | 0.716 |
| 15 | *Osteospermum moniliferum* L. | positive | 0.103 | 0.657 |
| 16 | *Sideroxylon inerme* L. | positive | 0.334 | 0.139 |
| 17 | *Cassine peragua* L. | positive | 0.086 | 0.711 |
| 18 | *Gymnosporia buxifolia* (L.) Szyszył*.* | positive | 0.349 | 0.121 |
| 19 | *Scolopia zeyheri* (Nees) Szyszył. | positive | 0.326 | 0.149 |
| 20 | *Osyris compressa* A.DC. | positive | 0.256 | 0.262 |
| Invasive alien plants | | | | |
| 21 | *Pinus pinaster* Aiton | negative | 0.308 | 0.174 |
| 22 | *Pinus radiata* D.Don | negative | 0.255 | 0.264 |
| 23 | *Cestrum laevigatum* Schltdl. | positive | 0.138 | 0.550 |
| 24 | *Acacia cyclops* G.Don | negative | 0.179 | 0.435 |
| 25 | *Acacia melanoxylon* R.Br. | positive | 0.009 | 0.972 |
| 26 | *Acacia saligna* (Labill.) Wendl | positive | 0.003 | 0.993 |
| 27 | *Acacia mearnsii* De Wild. | positive | 0.229 | 0.318 |
| 28 | *Eucalyptus camaldulensis* Dehnh. | negative | 0.001 | 0.998 |
| 29 | *Callistemon viminalis* (Sol. ex Gaertn.) G.Don | negative | 0.217 | 0.343 |
| 30 | *Leptospermum laevigatum* (Gaertn.) F.Muell. | positive | 0.310 | 0.171 |
