# Supplementary material for: Fire weather effects on flammability of indigenous and invasive alien plants in coastal fynbos and thicket shrublands (Cape Floristic Region)
Source: PeerJ. 2020 Nov 11;8:e10161. doi: 10.7717/peerj.10161 (PMC7666561; doi:10.7717/peerj.10161)
Supplement: Supplemental Information 3 — R software version 3.6.1 (R Development Core Team 2019) was used. Response variables were burn intensity, completeness of burn and time-to-ignition. Predictor variables were (i) fire weather (ii) fuel moisture, (iii) fuel load, (iv) vegetation groups (IAPs, invasive alien plants; Fyn, fynbos; and Thi, thicket) and (v) species as random factor. Burn intensity, completeness of burn and time-to-ignition were run using generalized linear mixed-effects models (gaussian family and identity function) and spontaneous ignition (binomial family and logit link function) using lme4 package. [file peerj-08-10161-s003.docx]

Supplemental Table S2

Formulae used in R software version 3.6.1 (R Development Core Team 2019) that assessed flammability in terms of the response variables (burn intensity, completeness of burn and time-to-ignition) respectively, in relation to the predictor variables (i) fire weather (ii) fuel moisture, (iii) fuel load, (iv) vegetation groups (IAPs, invasive alien plants; Fyn, fynbos; and Thi, thicket).and (v) species as random factor. Burn intensity, completeness of burn and time-to-ignition were run using generalized linear mixed-effects models (gaussian family and identity function) and spontaneous ignition (binomial family and logit link function) using *lme4* package.

| Response variables | Linear mixed model ['lmerMod'] |
| --- | --- |
| Log_BurnIntensity | > GLMM.1 <- glmer (Log_BurnIntensity ~ FireWeather + FuelMoisture + FuelLoad + VegGroup + (1\|Species), family = gaussian (identity), data = Dataset)  > summary (GLM.1) |
| Arcsine_Completenessofburn | > GLMM.2 <- glmer (Arcsine_Completeness.of.burn ~ FireWeather + FuelMoisture + FuelLoad + VegGroup + (1\|Species), family = gaussian (identity), data = Dataset)  > summary (GLM.2) |
| Squareroot_TimeToIgnition | > GLMM.3 <- glmer (Sqrt_TimeToIgnition ~ FireWeather + FuelMoisture + FuelLoad + VegGroup + (1\|Species), family = gaussian (identity), data = Dataset)  > summary (GLM.3) |
| SpontaneousIgnition | > GLMM.4 <- glmer (SpontaneousIgnition ~ FireWeather + FuelMoisture + FuelLoad + VegGroup + (1\|Species), family = binomial (logit), data = Dataset)  > summary (GLM.4) |
