# Supplementary material for: Fire weather effects on flammability of indigenous and invasive alien plants in coastal fynbos and thicket shrublands (Cape Floristic Region)
Source: PeerJ. 2020 Nov 11;8:e10161. doi: 10.7717/peerj.10161 (PMC7666561; doi:10.7717/peerj.10161)
Supplement: Supplemental Information 4 [file peerj-08-10161-s004.docx]

Supplemental Table S3

Analysis of Deviance Table (Type II Wald chi-square tests) of the generalized linear mixed-effects models and logistic regression fitted as in Supplemental Table S3.

| Log_BurnIntensity | Chisq Df Pr(>Chisq)  FireWeather 4.1168 1 0.04246 *  FuelMoisture 4.3764 1 0.03644 *  FuelLoad 5.6331 1 0.01762 *  VegGroup 8.0701 2 0.01768 * |
| --- | --- |
| Arcsine_Completenessofburn | Chisq Df Pr(>Chisq)  FireWeather 11.0550 1 0.0008845 ***  FuelMoisture 4.6153 1 0.0316873 *  FuelLoad 1.1265 1 0.2885249  VegGroup 5.7229 2 0.0571855 |
| Squareroot_TimeToIgnition | Chisq Df Pr(>Chisq)  FireWeather 20.9586 1 4.693e-06 ***  FuelMoisture 2.7578 1 0.096779  FuelLoad 9.2720 1 0.002327 **  VegGroup 9.5618 2 0.008388 ** |
| SpontaneousIgnition | Chisq Df Pr(>Chisq)  FireWeather 23.8176 1 1.059e-06 ***  FuelMoisture 4.4745 1 0.0344038 *  FuelLoad 2.6076 1 0.1063494  VegGroup 16.3775 2 0.0002778 *** |

Significance codes: *p<0.05, **p <0.01, ***p <0.001
